# Supplementary material for: Mortality among 5 to 19-year-olds in rural Mali
Source: PLOS Glob Public Health. 2025 Jan 21;5(1):e0004172. doi: 10.1371/journal.pgph.0004172 (PMC11750098; doi:10.1371/journal.pgph.0004172)
Supplement: S1 Table — (DOCX) [file pgph.0004172.s001.docx]

S1 **Table – Rates of mortality by age, sex and household characteristics among 5 to 9-year-olds and 10 to 19-year-olds**

| **Characteristic** | **Categories** | **Ages 5 to 9-years-old** | | | **Categories** | **Ages 10 to 19-years-old** | | |
| --- | --- | --- | --- | --- | --- | --- | --- | --- |
|  |  | **Deaths** | **Person time, years** | **Rate per 1,000 person years (95%CI)** |  | **Deaths** | **Person time, years** | **Rate per 1,000 person years (95%CI)** |
| **Age and sex** | Male, 5-7 years | 69 | 19,021.7 | 3.6 (2.9, 4.6) | Male, 10-14 years | 37 | 21,065.1 | 1.8 (1.3, 2.4) |
|  | Male, 8-9 years | 19 | 10,808.0 | 1.8 (1.1, 2.8) | Male, 15-19 years | 17 | 10,889.9 | 1.6 (1.0, 2.5) |
|  | Female, 5-7 years | 65 | 17,919.4 | 3.6 (2.8, 4.6) | Female, 10-14 years | 34 | 18,008.6 | 1.9 (1.3, 2.6) |
|  | Female, 8-9 years | 27 | 10,214.0 | 2.6 (1.8, 3.9) | Female, 15-19 years | 20 | 6,876.9 | 2.9 (1.9, 4.5) |
| **Ethnicity** | Dogon | 166 | 54,067.0 | 3.1 (2.6, 3.6) | Dogon | 103 | 53,022.8 | 1.9 (1.6, 2.4) |
|  | Fulani | 5 | 2,671.7 | 1.9 (0.8, 4.5) | Fulani | 4 | 2,607.0 | 1.5 (0.6, 4.1) |
|  | Other | 9 | 1,224.4 | 7.4 (3.8, 14.1) | Other | 1 | 1,210.7 | 0.8 (0.1, 5.9) |
| **Wealth quintile** | Wealthiest | 31 | 11,727.9 | 2.6 (1.9, 3.8) | Wealthiest | 19 | 11,852.7 | 1.6 (1.0, 2.5) |
|  | Wealthy | 45 | 10,111.9 | 4.5 (3.3, 6.0) | Wealthy | 8 | 9,285.6 | 0.9 (0.4, 1.7) |
|  | Middle | 35 | 11,145.6 | 3.1 (2.3, 4.4) | Middle | 16 | 10,255.5 | 1.6 (1.0, 2.5) |
|  | Poor | 33 | 11,314.1 | 2.9 (2.1, 4.1) | Poor | 24 | 10,952.9 | 2.2 (1.5, 3.3) |
|  | Poorest | 35 | 13,510.5 | 2.6 (1.9, 3.6) | Poorest | 41 | 14,280.7 | 2.9 (2.1, 3.9) |
|  | Unknown | 1 | 153.2 | 6.5 (0.9, 46.4) | Unknown | 0 | 213.1 | 0 |
| **Decision making contribution of women in household** | Contribute | 50 | 17,540.0 | 2.9 (2.2, 3.8) | Contribute | 35 | 18,343.9 | 1.9 (1.4, 2.7) |
|  | Do not contribute | 118 | 38,511.8 | 3.1 (2.6, 3.7) | Do not contribute | 69 | 36,635.8 | 1.9 (1.5, 2.4) |
|  | Unknown | 12 | 1,911.3 | 6.3 (3.6, 11.1) | Unknown | 4 | 1,860.9 | 2.1 (0.8, 5.7) |
| **Highest level of reading abilty among women in household** | Can read | 6 | 2,303.4 | 2.6 (1.2, 5.8) | Can read | 0 | 3,013.1 | 0 |
|  | Can partly read | 3 | 1,494.3 | 2.0 (0.6, 6.2) | Can partly read | 2 | 1,681.3 | 1.2 (0.3, 4.8) |
|  | Cannot read | 159 | 52,012.6 | 3.1 (2.6, 3.6) | Cannot read | 102 | 50,082.6 | 2.0 (1.7, 2.5) |
|  | Unknown | 12 | 2,152.7 | 5.6 (3.2, 9.8) | Unknown | 4 | 2,063.5 | 1.9 (0.7, 5.2) |
| **Highest level of schooling among women in household** | Schooling | 16 | 5,386.8 | 3.0 (1.8, 4.8) | Schooling | 4 | 6,495.0 | 0.6 (0.2, 1.6) |
|  | No schooling | 152 | 50,668.3 | 3.0 (2.6, 3.5) | No schooling | 100 | 48,447.5 | 2.1 (1.7, 2.5) |
|  | Unknown | 12 | 1,908.0 | 6.3 (3.6, 11.1) | Unknown | 4 | 1,898.0 | 2.1 (0.8, 5.6) |
| **Polygamy** | Monogamous | 78 | 27,565.1 | 2.8 (2.3, 3.5) | Monogamous | 44 | 26,549.0 | 1.7 (1.2, 2.2) |
|  | Polygamous | 90 | 27,597.7 | 3.3 (2.7, 4.0) | Polygamous | 59 | 27,227.7 | 2.2 (1.7, 2.8) |
|  | Unknown | 12 | 2,800.2 | 4.3 (2.4, 7.5) | Unknown | 5 | 3,063.8 | 1.6 (0.7, 3.9) |
| **Domestic violence** | Not tolerated | 30 | 13,201.6 | 2.3 (1.6, 3.3) | Not tolerated | 25 | 12,676.2 | 2.0 (1.3, 2.9) |
|  | Tolerated | 136 | 42,183.5 | 3.2 (2.7, 3.8) | Tolerated | 78 | 41,581.6 | 1.9 (1.5, 2.3) |
|  | Unknown | 14 | 2,578.0 | 5.4 (3.2, 9.2) | Unknown | 5 | 2,582.8 | 1.9 (0.8, 4.7) |
| **Water source** | Improved and treated | 20 | 10,037.3 | 2.0 (1.3, 3.1) | Improved and treated | 13 | 10,405.4 | 1.2 (0.7, 2.2) |
|  | Improved but untreated | 71 | 22,008.6 | 3.2 (2.6, 4.1) | Improved but untreated | 38 | 21,436.8 | 1.8 (1.3, 2.4) |
|  | Unimproved but treated | 11 | 4,863.8 | 2.3 (1.3, 4.1) | Unimproved but treated | 15 | 4,853.5 | 3.1 (1.9, 5.1) |
|  | Unimproved and untreated | 76 | 20,694.3 | 3.7 (2.9, 4.6) | Unimproved and untreated | 42 | 19,750.2 | 2.1 (1.6, 2.9) |
|  | Unknown | 2 | 359.1 | 5.6 (1.4, 22.3) | Unknown | 0 | 394.6 | 0 |
| **Sanitation** | Improved | 85 | 29,253.3 | 2.9 (2.3, 3.6) | Improved | 58 | 29,481.0 | 2.0 (1.5, 2.5) |
|  | Unimproved | 92 | 28,345.2 | 3.2 (2.6, 4.0) | Unimproved | 49 | 26,943.5 | 1.8 (1.4, 2.4) |
|  | Unknown | 3 | 364.5 | 8.2 (2.7, 25.5) | Unknown | 1 | 416.0 | 2.4 (0.3, 17.1) |
| **Roofing material** | Finished | 159 | 50,604.0 | 3.1 (2.7, 3.7) | Finished | 92 | 49,467.7 | 1.9 (1.5, 2.3) |
|  | Rudimentary | 14 | 5,180.6 | 2.7 (1.6, 4.6) | Rudimentary | 12 | 5,090.7 | 2.4 (1.3, 4.2) |
|  | Natural | 6 | 1,869.9 | 3.2 (1.4, 7.1) | Natural | 4 | 1,909.2 | 2.1 (0.8, 5.6) |
|  | Unknown | 1 | 308.6 | 3.2 (0.5, 23.0) | Unknown | 0 | 373.0 | 0 |
| **Wall material** | Finished | 86 | 32,234.3 | 2.7 (2.2, 3.3) | Finished | 54 | 31,677.3 | 1.7 (1.3, 2.2) |
|  | Rudimentary | 25 | 5,759.7 | 4.3 (2.9, 6.4) | Rudimentary | 15 | 5,639.8 | 2.7 (1.6, 4.4) |
|  | Natural | 68 | 19,057.7 | 3.6 (2.8, 4.5) | Natural | 35 | 18,628.0 | 1.9 (1.3, 2.6) |
|  | Unknown | 1 | 911.4 | 1.1 (0.2, 7.8) | Unknown | 4 | 895.5 | 4.5 (1.7, 11.9) |
| **Electricity** | No | 114 | 36,906.1 | 3.1 (2.6, 3.7) | No | 70 | 35,530.3 | 2.0 (1.6, 2.5) |
|  | Yes | 66 | 21,057.0 | 3.1 (2.5, 4.0) | Yes | 38 | 21,310.2 | 1.8 (1.3, 2.5) |
| **Primary cooking fuel** | Wood | 149 | 49,280.7 | 3.0 (2.6, 3.6) | Wood | 89 | 48,020.3 | 1.9 (1.5, 2.3) |
|  | Straw | 29 | 7,606.1 | 3.8 (2.6, 5.5) | Straw | 17 | 7,719.5 | 2.2 (1.4, 3.5) |
|  | Animal dung | 1 | 661.1 | 1.5 (0.2, 10.7) | Animal dung | 2 | 597.9 | 3.3 (0.8, 13.4) |
|  | Other | 1 | 415.1 | 2.4 (0.3, 17.1) | Other | 0 | 502.8 | 0 |
| **Food shortage in past 30 days** | No | 158 | 49,260.4 | 3.2 (2.7, 3.7) | No | 91 | 47,900.8 | 1.9 (1.5, 2.3) |
|  | Yes | 22 | 8,702.7 | 2.5 (1.7, 3.8) | Yes | 17 | 8,939.7 | 1.9 (1.2, 3.1) |
| **Livestock** | No | 13 | 3,690.4 | 3.5 (2.0, 6.1) | No | 3 | 2,769.5 | 1.1 (0.3, 3.4) |
|  | Yes | 167 | 54,272.7 | 3.1 (2.6, 3.6) | Yes | 105 | 54,071.0 | 1.9 (1.6, 2.4) |
| **Motorized transport** | No | 79 | 27,089.4 | 2.9 (2.3, 3.6) | No | 53 | 25,643.0 | 2.1 (1.6, 2.7) |
|  | Yes | 101 | 30,873.7 | 3.3 (2.7, 4.0) | Yes | 55 | 31,197.5 | 1.8 (1.4, 2.3) |
| **Nearest healthcare center, kilometers** | <2 | 26 | 10,556.6 | 2.5 (1.7, 3.6) | <2 | 20 | 11,019.2 | 1.8 (1.2, 2.8) |
|  | 2 - 4.99 | 53 | 15,309.2 | 3.5 (2.6, 4.5) | 2 - 4.99 | 28 | 14,273.3 | 2.0 (1.4, 2.8) |
|  | 5 - 6.99 | 43 | 13,926.7 | 3.1 (2.3, 4.2) | 5 - 6.99 | 22 | 12,363.2 | 1.8 (1.2, 2.7) |
|  | 7 - 9.99 | 36 | 11,315.7 | 3.2 (2.3, 4.4) | 7 - 9.99 | 18 | 11,557.8 | 1.6 (1.0, 2.5) |
|  | ≥10 | 22 | 6,855.0 | 3.2 (2.1, 4.9) | ≥10 | 20 | 7,627.1 | 2.6 (1.7, 4.1) |

CI, confidence interval
