# Supplementary material for: Mortality among 5 to 19-year-olds in rural Mali
Source: PLOS Glob Public Health. 2025 Jan 21;5(1):e0004172. doi: 10.1371/journal.pgph.0004172 (PMC11750098; doi:10.1371/journal.pgph.0004172)
Supplement: S2 Table — (DOCX) [file pgph.0004172.s002.docx]

**S2 Table – Rates of mortality by age and household characteristics among males and females aged 5 to 19 years**

| **Characteristic** | **Categories** | **Male** | | | **Female** | | |
| --- | --- | --- | --- | --- | --- | --- | --- |
|  |  | **Deaths** | **Person time, years** | **Rate per 1,000 person years (95%CI)** | **Deaths** | **Person time, years** | **Rate per 1,000 person years (95%CI)** |
| **Age** | 5-7 years | 69 | 19,021.7 | 3.6 (2.9, 4.6) | 65 | 17,919.4 | 3.6 (2.8, 4.6) |
|  | 8-9 years | 19 | 10,808.0 | 1.8 (1.1, 2.8) | 27 | 10,214.0 | 2.6 (1.8, 3.9) |
|  | 10-14 years | 37 | 21,065.1 | 1.8 (1.3, 2.4) | 34 | 18,008.6 | 1.9 (1.3, 2.6) |
|  | 15-19 years | 17 | 10,889.9 | 1.6 (1.0, 2.5) | 20 | 6,876.9 | 2.9 (1.9, 4.5) |
| **Ethnicity** | Dogon | 136 | 57,626.7 | 2.4 (2.0, 2.8) | 133 | 49,463.0 | 2.7 (2.3, 3.2) |
|  | Fulani | 3 | 2,846.2 | 1.1 (0.3, 3.3) | 6 | 2,432.5 | 2.5 (1.1, 5.5) |
|  | Other | 3 | 1,311.9 | 2.3 (0.7, 7.1) | 7 | 1,123.3 | 6.2 (3.0, 13.1) |
| **Wealth quintile** | Wealthiest | 25 | 12,445.9 | 2.0 (1.4, 3.0) | 25 | 11,134.7 | 2.2 (1.5, 3.3) |
|  | Wealthy | 25 | 10,362.8 | 2.4 (1.6, 3.6) | 28 | 9,034.7 | 3.1 (2.1, 4.5) |
|  | Middle | 30 | 11,636.2 | 2.6 (1.8, 3.7) | 21 | 9,764.9 | 2.2 (1.4, 3.3) |
|  | Poor | 24 | 12,145.0 | 2.0 (1.3, 2.9) | 33 | 10,122.0 | 3.3 (2.3, 4.6) |
|  | Poorest | 38 | 15,007.3 | 2.5 (1.8, 3.5) | 38 | 12,783.8 | 3.0 (2.2, 4.1) |
|  | Unknown | 0 | 187.5 | 0 | 1 | 178.8 | 5.6 (0.8, 39.7) |
| **Decision making contribution of women in household** | Contribute | 43 | 19,065.6 | 2.3 (1.7, 3.0) | 42 | 16,818.3 | 2.5 (1.8, 3.4) |
|  | Do not contribute | 92 | 40,668.9 | 2.3 (1.8, 2.8) | 95 | 34,478.7 | 2.8 (2.3, 3.4) |
|  | Unknown | 7 | 2,050.3 | 3.4 (1.6, 7.2) | 9 | 1,721.9 | 5.2 (2.7, 10.0) |
| **Highest level of reading ability among women in household** | Can read | 3 | 2,450.4 | 1.2 (0.4, 3.8) | 3 | 2,866.1 | 1.0 (0.3, 3.2) |
|  | Can partly read | 2 | 1,647.8 | 1.2 (0.3, 4.9) | 3 | 1,527.7 | 2.0 (0.6, 6.1) |
|  | Cannot read | 130 | 55,401.4 | 2.3 (2.0, 2.8) | 131 | 46,693.9 | 2.8 (2.4, 3.3) |
|  | Unknown | 7 | 2,285.1 | 3.1 (1.5, 6.4) | 9 | 1,931.1 | 4.7 (2.4, 9.0) |
| **Highest level of schooling among women in household** | Schooling | 13 | 5,856.1 | 2.2 (1.3, 3.8) | 7 | 6,025.7 | 1.2 (0.6, 2.4) |
|  | No schooling | 122 | 53,859.7 | 2.3 (1.9, 2.7) | 130 | 45,256.1 | 2.9 (2.4, 3.4) |
|  | Unknown | 7 | 2,068.9 | 3.4 (1.6, 7.1) | 9 | 1,737.1 | 5.2 (2.7, 10.0) |
| **Polygamy** | Monogamous | 60 | 29,418.8 | 2.0 (1.6, 2.6) | 62 | 24,695.3 | 2.5 (2.0, 3.2) |
|  | Polygamous | 74 | 29,213.3 | 2.5 (2.0, 3.2) | 75 | 25,612.2 | 2.9 (2.3, 3.7) |
|  | Unknown | 8 | 3,152.6 | 2.5 (1.3, 5.1) | 9 | 2,711.3 | 3.3 (1.7, 6.4) |
| **Domestic violence** | Not tolerated | 24 | 13,940.4 | 1.7 (1.2, 2.6) | 31 | 11,937.4 | 2.6 (1.8, 3.7) |
|  | Tolerated | 111 | 45,038.6 | 2.5 (2.0, 3.0) | 103 | 38,726.5 | 2.7 (2.2, 3.2) |
|  | Unknown | 7 | 2,805.8 | 2.5 (1.2, 5.2) | 12 | 2,355.0 | 5.1 (2.9, 9.0) |
| **Water source** | Improved and treated | 16 | 11,075.2 | 1.4 (0.9, 2.4) | 17 | 9,367.6 | 1.8 (1.1, 2.9) |
|  | Improved but untreated | 47 | 23,451.1 | 2.0 (1.5, 2.7) | 62 | 19,994.3 | 3.1 (2.4, 4.0) |
|  | Unimproved but treated | 15 | 5,166.3 | 2.9 (1.8, 4.8) | 11 | 4,551.0 | 2.4 (1.3, 4.4) |
|  | Unimproved and untreated | 64 | 21,716.1 | 2.9 (2.3, 3.8) | 54 | 18,728.4 | 2.9 (2.2, 3.8) |
|  | Unknown | 0 | 376.2 | 0 | 2 | 377.6 | 5.3 (1.3, 21.2) |
| **Sanitation** | Improved | 76 | 31,424.2 | 2.4 (1.9, 3.0) | 67 | 27,310.1 | 2.5 (1.9, 3.1) |
|  | Unimproved | 65 | 29,923.5 | 2.2 (1.7, 2.8) | 76 | 25,365.2 | 3.0 (2.4, 3.8) |
|  | Unknown | 1 | 437.0 | 2.3 (0.3, 16.2) | 3 | 343.5 | 8.7 (2.8, 27.1) |
| **Roofing material** | Finished | 116 | 54,129.3 | 2.1 (1.8, 2.6) | 135 | 45,942.3 | 2.9 (2.5, 3.5) |
|  | Rudimentary | 20 | 5,359.8 | 3.7 (2.4, 5.8) | 6 | 4,911.5 | 1.2 (0.5, 2.7) |
|  | Natural | 6 | 1,933.6 | 3.1 (1.4, 6.9) | 4 | 1,845.5 | 2.2 (0.8, 5.8) |
|  | Unknown | 0 | 362.1 | 0 | 1 | 319.5 | 3.1 (0.4, 22.2) |
| **Wall material** | Finished | 59 | 34,389.9 | 1.7 (1.3, 2.2) | 81 | 29,521.7 | 2.7 (2.2, 3.4) |
|  | Rudimentary | 23 | 5,973.6 | 3.9 (2.6, 5.8) | 17 | 5,425.8 | 3.1 (1.9, 5.0) |
|  | Natural | 56 | 20,447.1 | 2.7 (2.1, 3.6) | 47 | 17,238.6 | 2.7 (2.0, 3.6) |
|  | Unknown | 4 | 974.1 | 4.1 (1.5, 10.9) | 1 | 832.7 | 1.2 (0.2, 8.5) |
| **Electricity** | No | 51 | 22,650.6 | 2.3 (1.7, 3.0) | 93 | 33,302.2 | 2.8 (2.3, 3.4) |
|  | Yes | 91 | 39,134.2 | 2.3 (1.9, 2.9) | 53 | 19,716.6 | 2.7 (2.1, 3.5) |
| **Primary cooking fuel** | Wood | 114 | 52,275.5 | 2.2 (1.8, 2.6) | 124 | 45,025.4 | 2.8 (2.3, 3.3) |
|  | Straw | 27 | 8,273.3 | 3.3 (2.2, 4.8) | 19 | 7,052.4 | 2.7 (1.7, 4.2) |
|  | Animal dung | 1 | 688.9 | 1.5 (0.2, 10.3) | 2 | 570.2 | 3.5 (0.9, 14.0) |
|  | Other | 0 | 547.1 | 0 | 1 | 370.9 | 2.7 (0.4, 19.1) |
| **Food shortage in past 30 days** | No | 129 | 52,160.8 | 2.5 (2.1, 2.9) | 120 | 45,000.3 | 2.7 (2.2, 3.2) |
|  | Yes | 13 | 9,623.9 | 1.4 (0.8, 2.3) | 26 | 8,018.5 | 3.2 (2.2, 4.8) |
| **Livestock** | No | 8 | 3,453.2 | 2.3 (1.2, 4.6) | 8 | 3,006.7 | 2.7 (1.3, 5.3) |
|  | Yes | 134 | 58,331.6 | 2.3 (1.9, 2.7) | 138 | 50,012.1 | 2.8 (2.3, 3.3) |
| **Motorized transport** | No | 60 | 28,359.2 | 2.1 (1.6, 2.7) | 72 | 24,373.2 | 3.0 (2.3, 3.7) |
|  | Yes | 82 | 33,425.6 | 2.5 (2.0, 3.0) | 74 | 28,645.6 | 2.6 (2.1, 3.2) |
| **Nearest healthcare center, kilometers** | <2 | 20 | 11,804.8 | 1.7 (1.1, 2.6) | 26 | 9,771.0 | 2.7 (1.8, 3.9) |
|  | 2 - 4.99 | 43 | 16,116.5 | 2.7 (2.0, 3.6) | 38 | 13,466.0 | 2.8 (2.1, 3.9) |
|  | 5 - 6.99 | 31 | 14,252.1 | 2.2 (1.5, 3.1) | 34 | 12,037.8 | 2.8 (2.0, 4.0) |
|  | 7 - 9.99 | 27 | 12,083.1 | 2.2 (1.5, 3.3) | 27 | 10,790.3 | 2.5 (1.7, 3.6) |
|  | ≥10 | 21 | 7,528.3 | 2.8 (1.8, 4.3) | 21 | 6,953.8 | 3.0 (2.0, 4.6) |

CI, confidence interval
