# Supplementary material for: Mortality among 5 to 19-year-olds in rural Mali
Source: PLOS Glob Public Health. 2025 Jan 21;5(1):e0004172. doi: 10.1371/journal.pgph.0004172 (PMC11750098; doi:10.1371/journal.pgph.0004172)
Supplement: S3 Table — (DOCX) [file pgph.0004172.s003.docx]

**S3 Table – Age and household characteristics associated with mortality among males and females aged 5 to 19 years**

| **Characteristic** | **Categories** | **Males** | | | | **Females** | | | |
| --- | --- | --- | --- | --- | --- | --- | --- | --- | --- |
|  |  | **Univariate hazard ratio (95%CI)** | **p** | **Multivariate hazard ratio (95%CI)** | **p** | **Univariate hazard ratio (95%CI)** | **p** | **Multivariate hazard ratio (95%CI)** | **p** |
| **Age** | 5-7 years | 1.00 |  | 1.00 |  | 1.00 |  |  |  |
|  | 8-9 years | 0.59 (0.36, 0.98) | 0.040 | 0.60 (0.36, 0.98) | 0.043 | 1.02 (0.65, 1.60) | 0.939 |  |  |
|  | 10-14 years | 0.61 (0.39, 0.95) | 0.027 | 0.61 (0.39, 0.95) | 0.030 | 0.73 (0.47, 1.14) | 0.166 |  |  |
|  | 15-19 years | 0.55 (0.32, 0.95) | 0.031 | 0.56 (0.33, 0.97) | 0.040 | 1.15 (0.66, 2.00) | 0.632 |  |  |
| **Ethnicity** | Dogon | 1.00 |  |  |  | 1.00 |  | 1.00 |  |
|  | Fulani | 0.45 (0.10, 1.94) | 0.283 |  |  | 0.92 (0.41, 2.07) | 0.837 | 0.89 (0.39, 2.02) | 0.777 |
|  | Other | 0.97 (0.31, 3.00) | 0.961 |  |  | 2.30 (1.09, 4.86) | 0.029 | 2.48 (1.17, 5.25) | 0.017 |
| **Wealth quintile** | Wealthiest | 1.00 |  |  |  | 1.00 |  |  |  |
|  | Wealthy | 1.19 (0.67, 2.14) | 0.554 |  |  | 1.37 (0.78, 2.41) | 0.276 |  |  |
|  | Middle | 1.28 (0.74, 2.22) | 0.384 |  |  | 0.95 (0.52, 1.75) | 0.872 |  |  |
|  | Poor | 0.98 (0.53, 1.81) | 0.955 |  |  | 1.44 (0.84, 2.49) | 0.186 |  |  |
|  | Poorest | 1.26 (0.73, 2.19) | 0.407 |  |  | 1.32 (0.78, 2.25) | 0.304 |  |  |
|  | Unknown | - |  |  |  | - |  |  |  |
| **Decision making contribution of women in household** | Contribute | 1.00 |  |  |  | 1.00 |  |  |  |
|  | Do not contribute | 1.00 (0.68, 1.48) | 0.999 |  |  | 1.10 (0.76, 1.59) | 0.623 |  |  |
|  | Unknown | - |  |  |  | - |  |  |  |
| **Highest level of reading ability among women in household** | Can read | 1.00 |  |  |  | 1.00 |  |  |  |
|  | Can partly read | 0.99 (0.16, 5.93) | 0.988 |  |  | 1.85 (0.38, 9.09) | 0.450 |  |  |
|  | Cannot read | 1.92 (0.61, 6.05) | 0.268 |  |  | 2.62 (0.84, 8.19) | 0.098 |  |  |
|  | Unknown | - |  |  |  | - |  |  |  |
| **Highest level of schooling among women in household** | Schooling | 1.00 |  |  |  | 1.00 |  | 1.00 |  |
|  | No schooling | 1.02 (0.57, 1.82) | 0.945 |  |  | 2.43 (1.14, 5.20) | 0.022 | 2.49 (1.16, 5.36) | 0.019 |
|  | Unknown | - |  |  |  | - |  | - |  |
| **Polygamy** | Monogamous | 1.00 |  |  |  | 1.00 |  |  |  |
|  | Polygamous | 1.24 (0.86, 1.81) | 0.252 |  |  | 1.17 (0.83, 1.65) | 0.374 |  |  |
|  | Unknown | - |  |  |  | - |  |  |  |
| **Domestic violence** | Not tolerated | 1.00 |  |  |  | 1.00 |  |  |  |
|  | Tolerated | 1.43 (0.90, 2.27) | 0.130 |  |  | 1.03 (0.67, 1.57) | 0.908 |  |  |
|  | Unknown | - |  |  |  | - |  |  |  |
| **Water source** | Improved and treated | 1.00 |  | 1.00 |  | 1.00 |  |  |  |
|  | Improved but untreated | 1.38 (0.78, 2.44) | 0.264 | 1.34 (0.76, 2.37) | 0.307 | 1.70 (0.97, 2.98) | 0.065 |  |  |
|  | Unimproved but treated | 2.00 (0.97, 4.12) | 0.060 | 1.95 (0.94, 4.01) | 0.071 | 1.33 (0.59, 2.98) | 0.492 |  |  |
|  | Unimproved and untreated | 2.03 (1.15, 3.58) | 0.014 | 1.87 (1.06, 3.30) | 0.031 | 1.57 (0.89, 2.78) | 0.119 |  |  |
|  | Unknown | - |  | - |  | - |  |  |  |
| **Sanitation** | Improved | 1.00 |  |  |  | 1.00 |  |  |  |
|  | Unimproved | 0.90 (0.62, 1.29) | 0.555 |  |  | 1.22 (0.86, 1.71) | 0.261 |  |  |
|  | Unknown | - |  |  |  | - |  |  |  |
| **Roofing material** | Finished | 1.00 |  |  |  | 1.00 |  |  |  |
|  | Rudimentary | 1.74 (1.02, 2.97) | 0.041 |  |  | 0.42 (0.16, 1.06) | 0.067 |  |  |
|  | Natural | 1.44 (0.64, 3.25) | 0.377 |  |  | 0.74 (0.28, 1.96) | 0.543 |  |  |
|  | Unknown | - |  |  |  | - |  |  |  |
| **Wall material** | Finished | 1.00 |  | 1.00 |  | 1.00 |  |  |  |
|  | Rudimentary | 2.24 (1.22, 4.13) | 0.010 | 2.15 (1.18, 3.90) | 0.012 | 1.14 (0.63, 2.05) | 0.669 |  |  |
|  | Natural | 1.60 (1.09, 2.35) | 0.017 | 1.54 (1.04, 2.27) | 0.030 | 0.99 (0.69, 1.43) | 0.960 |  |  |
|  | Unknown | - |  | - |  | - |  |  |  |
| **Electricity** | No | 1.00 |  |  |  | 1.00 |  |  |  |
|  | Yes | 0.97 (0.67, 1.41) | 0.888 |  |  | 0.96 (0.68, 1.37) | 0.838 |  |  |
| **Primary cooking fuel** | Wood | 1.00 |  |  |  | 1.00 |  |  |  |
|  | Straw | 1.50 (0.93, 2.39) | 0.093 |  |  | 0.98 (0.59, 1.62) | 0.944 |  |  |
|  | Animal dung | 0.66 (0.09, 4.60) | 0.675 |  |  | 1.27 (0.31, 5.20) | 0.736 |  |  |
|  | Other | nc |  |  |  | 0.99 (0.14, 7.30) | 0.994 |  |  |
| **Food shortage in past 30 days** | No | 1.00 |  |  |  | 1.00 |  |  |  |
|  | Yes | 0.55 (0.30, 1.01) | 0.054 |  |  | 1.22 (0.80, 1.86) | 0.365 |  |  |
| **Livestock** | No | 1.00 |  |  |  | 1.00 |  |  |  |
|  | Yes | 1.01 (0.46, 2.24) | 0.975 |  |  | 1.04 (0.51, 2.13) | 0.904 |  |  |
| **Motorized transport** | No | 1.00 |  |  |  | 1.00 |  |  |  |
|  | Yes | 1.16 (0.81, 1.67) | 0.416 |  |  | 0.87 (0.62, 1.22) | 0.434 |  |  |
| **Nearest healthcare center, kilometers** | <2 | 1.00 |  |  |  | 1.00 |  |  |  |
|  | 2 - 4.99 | 1.57 (0.86, 2.86) | 0.139 |  |  | 1.06 (0.61, 1.81) | 0.844 |  |  |
|  | 5 - 6.99 | 1.28 (0.70, 2.35) | 0.421 |  |  | 1.06 (0.62, 1.81) | 0.841 |  |  |
|  | 7 - 9.99 | 1.31 (0.72, 2.41) | 0.377 |  |  | 0.94 (0.53, 1.67) | 0.830 |  |  |
|  | ≥10 | 1.64 (0.84, 3.19) | 0.145 |  |  | 1.13 (0.62, 2.06) | 0.683 |  |  |

Children and adolescents with missing covariate data were included in analyses but hazard ratios for missing categories are not reported. CI, confidence interval; nc, non-calculable
