# Supplementary material for: Mortality among 5 to 19-year-olds in rural Mali
Source: PLOS Glob Public Health. 2025 Jan 21;5(1):e0004172. doi: 10.1371/journal.pgph.0004172 (PMC11750098; doi:10.1371/journal.pgph.0004172)
Supplement: Attachment — Submitted filename: PGPH-D-23-01206_reviewer, Mali, older adolescent mortality.pdf [file pgph.0004172.s004.pdf]

PLOS Global Public Health

Older child and adolescent mortality in rural Mali

--Manuscript Draft--

**Manuscript Number:**	PGPH-D-23-01206
**Article Type:**	Research Article
**Full Title:**	Older child and adolescent mortality in rural Mali
**Short Title:**	Older child and adolescent mortality in rural Mali
**Corresponding Author:**	Jenny Liu, PhD, MPP<br>University of California San Francisco<br>San Francisco, CA UNITED STATES
**Order of Authors:**	Jenny X. Liu, PhD, MPP
	Yacouba Samake, MD
	Oumar Tolo, MSc
	Emily Treleaven, PhD
	Belco Poudiougou, MD
	Caroline Whidden, MSc
	Ari Johnson, MD
	Kassoum Kayentao, MD
	David C. Boettiger, PhD
**Keywords:**	Child; adolescent; mortality; Household; Survey; Low-middle income
**Abstract:**	The unique healthcare needs of older children (5-9 years) and adolescents (10-19 years) in low- and middle-income countries have been largely neglected. We generated estimates of older child and adolescent mortality in rural Mali, a setting with high under-five mortality, and aimed to define associated individual and household risk factors.<br>We analysed baseline household survey data from the ProCCM trial (NCT02694055) conducted in Bankass District, Mali collected in December 2016 and January 2017. Deaths in the preceding five years, household information, and women's birth histories were documented. Factors associated with older child and adolescent mortality were analysed using Cox regression.<br>Our study population comprised of 23,485 children and 17,910 adolescents from 7,720 households. The child and adolescent mortality rates were 3.10 and 1.90 deaths per 1,000 person-years, respectively. Mortality rates were similar among males and females during childhood, but grew increasingly divergent in adolescence (1.69 and 2.17 per 1,000 person-years, respectively). Children in households with untreated water had a higher risk of death than those in households with treated water. Adolescents living in the poorest households had a higher risk of death than those in the wealthiest, and adolescents in households in which no women received schooling had a higher risk of death than those in which women had some schooling.<br>The risk of mortality is especially acute among female adolescents compared to their male counterparts, with low access to education for women being a strong contributing factor.
**Additional Information:**	
**Question**	**Response**
**Financial Disclosure**<br><br>Enter a financial disclosure statement that	The ProCCM trial is funded with resources received by Muso though unrestricted funding as well as dedicated research funding from Child Relief International Foundation, Grand Challenges Canada, Johnson & Johnson Foundation and USAID Development Innovation Ventures. Child Relief International Foundation serves as the nonlegal sponsor of the trial. Funders were not involved in the design of this analysis,

Powered by Editorial Manager® and ProduXion Manager® from Aries Systems Corporation

describes the sources of funding for the work included in this submission and the role the funder(s) played. This includes grants and any commercial funding of the work or authors.<br><br>This statement will be typeset if the manuscript is accepted for publication.<br><br>*Please review the submission guidelines and the instructions link below for detailed requirements and guidance.*	the collection, analysis, or interpretation of data, or the writing the manuscript.
**Competing Interests**<br><br>On behalf of all authors, disclose any competing interests that could be perceived to bias this work.<br><br>This statement will be typeset if the manuscript is accepted for publication.<br><br>*Please review the instructions link below and PLOS Global Public Health's competing interests policy to determine what information must be disclosed at submission.*	All authors report no competing interests.
**Data Availability**<br><br>Before publication, Authors are required to make fully available and without restriction all data underlying their findings. Please see our PLOS Data Policy page for detailed information on this policy.<br><br>A **Data Availability Statement**, detailing where the data can be accessed, is required at first submission. Insert your Data Availability Statement in the box below.<br><br>Please see the data reporting section of	Muso Inc. (https://www.musohealth.org/) own the data used for this study and granted access to the authors. These data are not publicly available to maintain participant privacy but de-identified data will be made available to external researchers upon reasonable request. Please direct enquiries to the corresponding author.

our submission guidelines for instructions on what you need to include in your Data Availability Statement.<br><br>This statement will be typeset if the manuscript is accepted for publication.<br><br>*PLOS allows rare exemptions to address legal and ethical concerns. If you have legal or ethical restrictions, please use the box below to detail these in full sentences for the Journal team to consider.*	

Manuscript

Click here to access/download;Manuscript;Manuscript 20230620.docx

Older child and adolescent mortality in rural Mali

Jenny X. Liu[a], PhD, MPP, Jenny.Liu2@ucsf.edu

Yacouba Samake[b], MD, yacousamake@gmail.com

Oumar Tolo[b], MSc, otolo@musohealth.org

Emily Treleaven[c], PhD, treleav@umich.edu

Belco Poudiougou[b], MD, bpoudiougou@musohealth.org

Caroline Whidden[b,d], MSc, cwhidden@musohealth.org

Ari Johnson[b,e], MD, ari.johnson@ucsf.edu

Kassoum Kayentao[b,g], MD, kayentao@icermali.org

David C. Boettiger[a,h,I], PhD, dboettiger@kirby.unsw.edu.au

[a] Institute for Health and Aging, School of Nursing, University of California San Francisco; San Francisco, CA, U.S.

[b] Muso Health; Bamako, Mali

[c] Survey Research Center, Institute for Social Research, University of Michigan; Ann Arbor, Michigan, U.S.

[d] Department of Disease Control, London School of Hygiene and Tropical Medicine; London, U.K.

[e] Department of Medicine, University of California, San Francisco; San Francisco, CA, U.S.

[g] Malaria Research and Training Center, University Sciences, Techniques, and Technologies of Bamako; Bamako, Mali

[h] The Kirby Institute, University of New South Wales; Sydney, Australia

[i] Biostatistics Excellence Centre, Chulalongkorn University; Bangkok, Thailand

* Correspondence to: **Dr. Jenny X. Liu**

Institute for Health and Aging, School of Nursing

University of California San Francisco; San Francisco, CA, U.S.
Email: Jenny.Liu2@ucsf.edu
P +1 415 502 5200

Abstract

The unique healthcare needs of older children (5-9 years) and adolescents (10-19 years) in low- and middle-income countries have been largely neglected. We generated estimates of older child and adolescent mortality in rural Mali, a setting with high under-five mortality, and aimed to define associated individual and household risk factors.

We analysed cross-sectional baseline household survey data from the ProCCM trial (NCT02694055) conducted in Bankass District, Mali collected in December 2016 and January 2017. Deaths in the preceding five years, household information, and women's birth histories were documented. Factors associated with older child and adolescent mortality were analysed using Cox regression.

Our study population comprised of 23,485 children and 17,910 adolescents from 7,720 households. The child and adolescent mortality rates were 3.10 and 1.90 deaths per 1,000 person-years, respectively. Mortality rates were similar among males and females during childhood, but grew increasingly divergent in adolescence (1.69 and 2.17 per 1,000 person-years, respectively). Children in households with untreated water had a higher risk of death than those in households with treated water. Adolescents living in the poorest households had a higher risk of death than those in the wealthiest, and adolescents in households in which no women received schooling had a higher risk of death than those in which women had some schooling.

The risk of mortality is especially acute among female adolescents compared to their male counterparts, with low access to education for women being a strong contributing factor.

Introduction

Despite global commitments to reducing the burden of disease among low- and middle-income countries (LMICs)[1], older children (age 5-9 years) and adolescents (age 10-19 years) are populations that have often been neglected by researchers and policymakers alike. Middle childhood and adolescence are key development periods in the life course, with health outcomes in this period shaping health, educational attainment, socio-economic status, and well-being in adulthood. Renewed efforts to focus on the needs of adolescents in LMICs have spurred a recent surge in research on their mental health [2], reproductive health [3], socioeconomic wellbeing [4], and schooling [5], often in the context of child development during the transition to adulthood.

awkward wording

Yet, the number of these studies constitutes only a small fraction of the literature compared to other age groups, particularly children under five years of age. Very few studies estimate mortality rates in older childhood and adolescence [6,7]; vital registration data can be of poor quality, particularly in LMICs where mortality rates are elevated. Using modelling approaches to address data quality challenges, an analysis of cause of death among of older children and adolescents in LMICs suggests that injuries and non-communicable disease comprise a greater proportion of cause of death than for children under five[8]. Correspondingly, these age groups are absent in many population health surveys in LMICs. Demographic Health Surveys, for example, include older female adolescents (age 15-19) in reference to fertility, reproductive, and maternal health, and nothing about male adolescents. UNICEF's Multiple Indicators Cluster Survey has included education, development, labour, and disciplining for those aged 5-17, and more recently added questions about functional health [9]. A limited number of targeted and ad hoc population-level surveys in LMICs have detailed measures of physical or mental health for middle childhood and adolescents [see for example 10], including the 2021 Global Youth Survey [11]. Yet, recent analyses show that communicable diseases continue to drive mortality into middle childhood while mental health and physical injuries become increasingly relevant in adolescence [8]. Little information exists to understand the overall health profiles of older children and adolescents in many settings globally, and

particularly in many of the poorest countries, though a growing body of studies have called attention to an array of multi-level risk and protective factors [12–14].

The current analysis forms part of a larger study evaluating the effect of proactive case detection by community health workers (CHW) on under-five mortality in the Bankass health district [15]. Bankass is situated in the Mopti region of Mali, an area that relies heavily on agriculture and that serves as an important crossroad between the country's north, south, and bordering countries [16]. It is one of the poorest regions of Mali and has one of the country's highest burdens of under-five child mortality [17,18]. However, little is known about the health outcomes and needs of older children and adolescents in this high morbidity, high mortality setting. As such, we aimed to (i) describe the trends in mortality for older children and adolescents, and (ii) identify the associated individual and household risk factors associated with older child and adolescent mortality. Our findings are discussed in light of the dearth of population-level data on older children and adolescents, and highlight areas of critical need for further research and health programming to improve survival among this group.

Methods

Household Survey Data

We analysed household census and survey data collected cross-sectionally at the baseline of a three-year cluster randomized controlled trial conducted in 137 village-clusters distributed across seven of the 22 primary health center (PHC) catchment areas in Bankass (Kanibonzon, Ende, Dimbal, Doundé, Soubala, Koulongon, and Lessagou). The study area has a population of approximately 100,000 people. The trial primarily aims to determine whether door-to-door proactive case detection by CHWs reduces under-five mortality compared to passive, site-based CHW care offered under the standard Integrated Community Case Management protocol [19]. The trial is registered with ClincialTrials.gov (NCT02694055). Ethical approval was obtained from the Ethics Committee of the Faculty of Medicine, Pharmacy and Dentistry, University of Bamako (2016/03/CE/FMPOS). The University of California, San Francisco

exempted secondary analysis of the deidentified trial data from ethical approval. All study participants provided written informed consent prior to enrolling in the study, which included the baseline survey. None of the authors had access to information that could identify individuals during or after data collection. Further details on the trial protocol are available elsewhere [15].

As a part of the trial procedures, a census was conducted among all households in the study area at baseline and updated every 12 months thereafter during the three-year study period. All households were censused between December 2016 and January 2017, just before the launch of the intervention, to enumerate all permanent residents (present more than 50% of the time in the past year). The census included a household roster to collect the age, date of birth, and sex of permanent residents, as well as information about deaths in each household in the past five years. One female member of the household was invited to complete a household survey of socio-economic factors. All women in the household aged 15-49 years (i.e., women of reproductive age) with no plans to leave the study area during the trial period who provided written informed consent were eligible to partake in the baseline survey. No children were surveyed. Surveys were adapted from the Malian Demographic and Health Surveys [18]. Women were given a reading test to assess literacy and asked if they contributed to household decision-making, if their husband had more than one wife or partner, and if they felt their husband hitting or beating them was justified under certain circumstances. Geographic coordinates were collected using global positioning technology. We supplemented the baseline survey data with information to accurately calculate the outcome from the 12-month follow-up survey, administered from February to April 2018, which used the same structure as the baseline survey but added details on women's lifetime birth histories (i.e., probes to distinguish between live and still births, clarification of multiple births, and greater precision on birth dates). This additional data enabled us to correct any missing data in the birth histories recorded at baseline. Survey instruments for the baseline and 12-month follow-up surveys are available for reference elsewhere [17].

word "elsewhere" not needed

Inclusion Criteria

Children (age 5 to 9 years) and adolescents (age 10 to 19 years) included in the analysis were those reported in lifetime birth histories by women who participated in the baseline and 12-month follow-up surveys as additional birth history data collected at 12 months (see *Household Survey Data* above) was required to accurately calculate baseline mortality rates. Based on dates of birth, all persons who were or would have been aged 5 to 19 years for ≥1 day in the five years prior to baseline were included in our analyses.

Variable Definitions

Household wealth was defined in quintiles using a principal components analysis of household possession of durable goods [20]. Women were considered to contribute to household decisions if they indicated sole or joint participation in decision-making in any of four domains—her own healthcare, visiting family or relatives, use of husband/partner's earnings, and household purchasing. Literacy was categorized based on the highest level of reading ability among women surveyed within the household (any, partial, full). Schooling was categorized as having had any formal schooling versus none. Polygamy was defined as a survey respondent in the household indicating that her husband/partner had more than one wife/partner. Women were considered to have tolerant views towards spousal violence if they indicated that their husband hitting or beating them was justified under any of seven circumstances evaluated in the survey (going out without telling, neglecting the children, arguing, refuses sex, burns food, uses contraception covertly, argues with partners' parents).

We used the World Health Organization definitions of improved (e.g., household connections, public standpipes, boreholes, protected dug wells, protected springs and rainwater collection) and unimproved (e.g., unprotected wells or springs, surface water, vendor-or tanker truck-provided) water supply and sanitation [21]. We combined this classification of facilities with self-reported application of water treatment for safer drinking (e.g., boiling, adding sterilizing chemicals, filtering, and solar disinfection) to

create four classifications: improved and treated; improved, but untreated; unimproved, but treated; unimproved and untreated. Roofing, wall, and flooring materials were defined as finished, rudimentary, or natural as per DHS definitions [22]. Distance to the nearest primary health center was defined as the Euclidean distance from the household village to the closest primary health center. All distance calculations were conducted in QGIS Version 3.4.6-Madeira (QGIS Development Team (2019), QGIS Geographic Information System, Open Source Geospatial Foundation Project, http://qgis.osgeo.org).

Statistical Analysis

The primary outcome was death. Household-level factors analyzed in older children and adolescents were ethnicity, wealth quintile, decision making contribution of women in household, highest level of reading ability among women in household, schooling among women in household, polygamy, domestic violence tolerance, water source, sanitation method, roofing material, wall material, flooring material, electricity access, primary cooking fuel, recent food shortage, livestock ownership, access to motorized transport, and distance of household from nearest healthcare center.

Cumulative probability of death from age 5 to 19 years was plotted for males and females separately using Kaplan-Meier curves. Cox regression models were used to evaluate factors associated with death. In our child models, children were considered at risk of death from the date they turned five years old or at the beginning of the five-year period prior to baseline if aged five to nine years at the time. Follow-up was censored on the day children turned ten years old or at the time their mother completed the baseline survey, whichever came first. In our adolescent models, adolescents were considered at risk of death from the date they turned ten years old or at the beginning of the five-year period prior to baseline if aged ten to 19 years at the time. Follow-up was censored on the day children turned 20 years old or at the time their mother completed the baseline survey, whichever came first.

Regression models were estimated separately by age group (i.e., older children vs. adolescents) and by sex (i.e., males vs. females). All regression analyses were adjusted for clustering at the household level

using robust standard errors. Covariates were subject to bivariate analysis and those with an adjusted p-value ≤ 0.05 were retained in our multivariate models. Age (years) and sex were included *a priori* in all multivariate models. Children and adolescents with missing covariate data were included in analyses but hazard ratios (HRs) for missing categories are not reported.

We further provide summary statistics for marriage, contraception, and pregnancy indicators among female adolescents of reproductive age (15-19 years) included in the women's survey to investigate possible additional risk factors for female adolescent mortality.

Analyses were conducted with Stata 16 (Stata Corp., College Station, Texas).

Results

Study population characteristics

Of 15,839 households censused at baseline, 268 (1.7%) were excluded because they did not have baseline and 12-month follow-up survey data, 2,632 (16.6%) did not include a woman of reproductive age, and 5,219 (33.0%) did not include an older child or adolescent in the past five years. This left a final sample of 7,720 households, 23,485 older children (12,060 male and 11,425 female), and 17,910 adolescents (9,823 male and 8,087 female). Table 1 presents further details of our study population.

Death by age and sex

Figure 1 shows that males and females died at a similar rate between the ages of 5 to 8 years. From 9 years of age, the probability of death among females became greater than that of similarly aged males and this gap continued to widen throughout adolescence.

Factors associated with death in older children

In the five years prior to baseline, a total of 180 child deaths occurred at a rate of 3.10 per 1,000 person years (2.95 and 3.27 per 1,000 person years for males and females, respectively; further disaggregated rates given in Tables A1 and A2). Table 2 shows that children living in households with an untreated water source had a higher risk of death than children living in households with a treated water source (adjusted hazard ratio [aHR] 1.61 for improved but untreated versus improved and treated, 95% confidence interval [CI] 0.98-2.66, p=0.061 and aHR 1.83 for unimproved and untreated versus improved and treated, 95% CI 1.11-3.02, p=0.018). Estimates for these risk factors were driven by associations among male older children in bi- and multi-variate models (see Table A3), in which household infrastructural features—water source and wall materials—emerged as contributing factors.

Factors associated with death in adolescents (ages 10-19 years)

In the five years prior to baseline, a total of 108 adolescent deaths occurred at a rate of 1.90 per 1,000 person years (1.69 and 2.17 per 1,000 person years for males and females, respectively; further disaggregated rates given in Tables A1 and A2). Table 2 shows that adolescents living in households in the poorest wealth quintile had a higher risk of death than those living in the wealthiest households (aHR 1.76 for poorest versus wealthiest, 95% CI 0.99-3.14, p=0.055). Adolescents living in households in which women had received no schooling had a higher risk of death than those living in households in which women had received schooling (aHR 3.52 for no schooling versus any schooling, 95% CI 1.29-9.62, p=0.014). Maternal education emerged as a strong contributing risk factor among females (see Table A3), driving the overall association among all adolescents.

Marriage, contraception, and pregnancy in female adolescents of reproductive age (15-19 years)

Of 3,143 females aged 15-19 years in our study population, 543 (17.3%) had information available on marriage, contraception, and pregnancy (Table 3). Of these, 147 (27.1%) were married with a median age at first marriage of 18.0 years (IQR 16.0-18.0). Overall, 10 (3.7%) reproductive age female

adolescents were using a method of contraception. Among those who were married, 1 (0.7%) was using a method of contraception. Thirteen (2.4%) reproductive age female adolescents were pregnant at the time of the survey (6, 1.1%) or had previously given birth (7, 1.3%).

Discussion

Amid the dearth of population health data on older children and adolescents in LMICs, our measurements contribute to filling this gap, highlighting the severity of mortality as children continue to age into adolescence and early adulthood in rural Mali. From our trial's baseline census of all

suggest more specificity here: although mortality rates generally decline from early childhood to later childhood and adolescence

households residing in our study area, we find that, although the mortality rate generally declines with age, child and adolescent mortality remain high. Compared to 2016 estimates of global all-cause mortality rates of 1, 0.7, and 1.2 deaths per 1000 person years for children ages 5-9, 10-14, and 15-19, respectively [7], mortality among older children in Bankass is three times as high, while that among adolescent is about twice as high. Older child (5-9 years) mortality in our study is elevated compared to the African regional average (3.1 vs. 2.0 deaths per 1000 person years), but adolescent mortality is comparable (1.9 deaths per 1000 person years among 10-19 year-olds in our study vs. 1.5 and 2.6 per 1000 person years among 10-14 and 15-19 year-olds, respectively, in the African Region) [7]. Notably, the risk of mortality is especially acute among female adolescents compared to their male counterparts; while indicators of poverty (poor infrastructure, low wealth) contributed overall, low maternal education within the household was a uniquely contributing factor for females. This difference in mortality by sex reverses the trend observed in our earlier analysis of under-fives in which mortality among males was greater than females [17], the latter of which is consistent with biological mechanisms [23] and national and international data where sex selection at birth is less prevalent [24]. Of available global

high-income

average trends, including among high income countries and LMICs, the risk of death among males increases overall and in relation to females during adolescence; only within African and Southeast Asian

countries, where many of the poorest countries are located, do females experience greater mortality into young adulthood [25], similar to what we observe in rural Mali.

This trend reversal and disparity underscores the importance of the confluence of risk factors, such as early marriage, childbearing, access to reproductive health, and gender-based violence, among many other inter-related dimensions of gender inequities (e.g., literacy, education, economic and financial dependence) that uniquely affect adolescent girls and young women in LMICs [26]. Accordingly, we also find that low maternal education as a unique driving risk factor among females, but it is unclear if

Rework this sentence. The way it is worded, you are implying that maternal education causes increased risk of death

maternal education itself causes elevated risk of death or rather is indicative of larger gender inequalities that disadvantage women. Our data do not permit a more detailed analysis of individual-level risk factors associated with female adolescent mortality, a limitation for understanding the significance of this result. Further, in comparison to Mali nationally, a lower percentage of females 15-19 in Bankass were married (27.1% vs. 39.7%), a surprising finding given the extreme poverty and poor health indicators for the region which are often strongly associated with early marriage. However, our sample of females age 15-19 in Bankass is small, as a larger number of older adolescent females than males were reported by their household to migrate out of the study area, which may be for reasons of marriage or employment, and also limits measurement accuracy. More research with a larger, in-depth survey of individuals is needed to fully assess and disentangle these risk factors for both males and females in our study population.

Our findings should be interpreted considering several additional limitations. Mortality was based on household members' recall of deaths in the preceding five years, which may be subject to recall errors, particularly for deaths occurring early in the five-year period. Elucidation of individual and household risk factors were further limited by the scope of survey questions, which do not represent a comprehensive assessment, particularly for individual-level factors (e.g., physical violence) that other studies have found to importantly contribute to older child and adolescent mortality [7,8]. Finally, we are unable to further ascribe the causes and contributors to mortality, which may require more in-depth verbal autopsy approaches in the absence of vital registration data.

Conclusion

Limitations notwithstanding, our data is unique among population health surveys in its comprehensive census of a large population in a resource-poor area of the world with one of the highest burdens of child mortality. Our results point to the critical need for further research and health programming to improve survival among older children and adolescents—in particular, a better accounting and measurement of their health outcomes and associated risk factors. The lack of attention to this gap does little to improve global health equity and must be urgently addressed, beginning with inclusion of these groups in population health research.

Abbreviations

CHW - community health workers

HRs - hazard ratios

LMICs - low- and middle-income countries

PHC - primary health center

Declarations

Acknowledgements

We are grateful to all study participants. We would also like to acknowledge Djoumé Diakité, Youssouf Keita, Aminata Konipo, and Seydou Sidibé of Muso for their role in implementing the trial and assuring adherence to protocol. This work was presented in preliminary form at the Health Systems Research conference in Bogota (Oct 31-Nov 4, 2022).

Ethics approval and consent to participate

The trial is registered with ClincialTrials.gov (NCT02694055). Ethical approval was obtained from the Ethics Committee of the Faculty of Medicine, Pharmacy and Dentistry, University of Bamako (2016/03/CE/FMPOS). The University of California, San Francisco exempted secondary analysis of the deidentified trial data from ethical approval. All participants provided written informed consent prior to enrolling in the study, which included the baseline survey.

Availability of data and materials

Muso Inc. (https://www.musohealth.org/) own the data used for this study and granted access to the authors. These data are not publicly available to maintain participant privacy but de-identified data will be made available to external researchers upon reasonable request. Please direct enquiries to the corresponding author.

Competing interests

All authors report no competing interests.

Funding

The trial is funded with resources received by Muso though unrestricted funding as well as dedicated research funding from Child Relief International Foundation, Grand Challenges Canada, Johnson & Johnson Foundation and USAID Development Innovation Ventures. Child Relief International Foundation serves as the nonlegal sponsor of the trial. Funders were not involved in the design of this analysis, the collection, analysis, or interpretation of data, or the writing the manuscript.

References

1. Desa U. *Transforming Our World: The 2030 Agenda for Sustainable Development.* United Nations; 2016.

2. Kieling C, Baker-Henningham H, Belfer M, et al. Child and adolescent mental health worldwide: evidence for action. *The Lancet*. 2011;378(9801):1515-1525. doi:10.1016/S0140-6736(11)60827-1

3. Melesse DY, Mutua MK, Choudhury A, et al. Adolescent sexual and reproductive health in sub-Saharan Africa: who is left behind? *BMJ Glob Health*. 2020;5(1):e002231. doi:10.1136/bmjgh-2019-002231

4. Bergstrom K, Ozler B. *Improving the Well-Being of Adolescent Girls in Developing Countries*. World Bank; 2021. doi:10.1596/1813-9450-9827

5. Murphy-Graham E, Lloyd C. Empowering adolescent girls in developing countries: The potential role of education. *Policy Futur Educ*. 2016;14(5):556-577. doi:10.1177/1478210315610257

6. Fadel SA, Boschi-Pinto C, Yu S, et al. Trends in cause-specific mortality among children aged 5–14 years from 2005 to 2016 in India, China, Brazil, and Mexico: an analysis of nationally representative mortality studies. *The Lancet*. 2019;393(10176):1119-1127. doi:10.1016/S0140-6736(19)30220-X

7. Strong KL, Pedersen J, Johansson EW, et al. Patterns and trends in causes of child and adolescent mortality 2000–2016: setting the scene for child health redesign. *BMJ Glob Health*. 2021;6(3):e004760. doi:10.1136/bmjgh-2020-004760

8. Liu L, Villavicencio F, Yeung D, et al. National, regional, and global causes of mortality in 5–19-year-olds from 2000 to 2019: a systematic analysis. *Lancet Glob Health*. 2022;10(3):e337-e347. doi:10.1016/S2214-109X(21)00566-0

9. UNICEF. Tools - UNICEF MICS. Multiple Cluster Indicators Survey. Accessed September 1, 2022. https://mics.unicef.org/tools#survey-design

10. Population Council. *Survey of Young People in Egypt*. Population Council; 2010.

11. Global Youth Survey for the World Youth Report 2021: Improving Youth Wellbeing and Mental Health. Global Youth Survey. Accessed September 1, 2022. https://www.un.org/development/desa/youth/news/2020/12/wyr2021-survey/

12. Patton GC, Sawyer SM, Santelli JS, et al. Our future: a Lancet commission on adolescent health and wellbeing. *The Lancet*. 2016;387(10036):2423-2478. doi:10.1016/S0140-6736(16)00579-1

13. Mmari K, Sabherwal S. A Review of Risk and Protective Factors for Adolescent Sexual and Reproductive Health in Developing Countries: An Update. *J Adolesc Health*. 2013;53(5):562-572. doi:10.1016/j.jadohealth.2013.07.018

14. Lassi ZS, Salam RA, Das JK, Wazny K, Bhutta ZA. An unfinished agenda on adolescent health: Opportunities for interventions. *Semin Perinatol*. 2015;39(5):353-360. doi:10.1053/j.semperi.2015.06.005

15. Whidden C, Treleaven E, Liu J, et al. Proactive community case management and child survival: protocol for a cluster randomised controlled trial. *BMJ Open*. 2019;9(8). doi:10.1136/bmjopen-2018-027487

16. Central Census Bureau (Mali). Mali Population and Housing Census 2009. Published online June 26, 2019. Accessed September 1, 2022. https://ghdx.healthdata.org/record/mali-population-and-housing-census-2009

17. Boettiger DC, Treleaven E, Kayentao K, et al. Household factors and under-five mortality in Bankass, Mali: results from a cross-sectional survey. *BMC Public Health*. 2021;21(1):244. doi:10.1186/s12889-021-10242-9

18. Cellule de Planification et de Statistique, Institut National de la Statistique, Centre d'Études et d'Information Statistiques, ICF International. *Mali Demographic and Health Survey, 2012-2013*.; 2014. Accessed October 9, 2018. https://dhsprogram.com/pubs/pdf/FR286/FR286.pdf

19. Ministere de la Sante et de l'Hygiene Publique. *Soins Essentiels Dans La Communaute: Guide de Mise En Oeuvre*. Direction Nationale de la Sante; 2015. https://www.unicef.org/mali/media/2291/file/PSN%202026-2020.pdf

20. Filmer D, Pritchett L. Estimating wealth effects without expenditure data--or tears: an application to educational enrollments in states of India. *Demography*. 2001;38:115-132.

21. WHO U. *Progress on Drinking Water and Sanitation. Joint Monitoring Programme for Water Supply and Sanitation (JMP)*. Geneva: WHO, UNICEF; 2012.

22. Croft TN, Marshall AM, Allen CK. Guide to DHS Statistics. DHS-7: The Demographic and Health Surveys Program. *ICF Rockv*. Published online 2018.

23. Waldron I. Sex differences in human mortality: The role of genetic factors. *Soc Sci Med*. 1983;17(6):321-333. doi:10.1016/0277-9536(83)90234-4

24. Sawyer CC. Child Mortality Estimation: Estimating Sex Differences in Childhood Mortality since the 1970s. *PLOS Med*. 2012;9(8):e1001287. doi:10.1371/journal.pmed.1001287

25. Patton GC, Coffey C, Sawyer SM, et al. Global patterns of mortality in young people: a systematic analysis of population health data. *The Lancet*. 2009;374(9693):881-892. doi:10.1016/S0140-6736(09)60741-8

26. UNAIDS. *The Gap Report 2014: Adolescent Girls and Young Women*. Joint United Nations Programme on HIV/AIDS (UNAIDS) Accessed September 1, 2022. https://www.unaids.org/en/resources/documents/2014/Adolescentgirlsandyoungwomen

Table 1 – Household characteristics for children and adolescents in Bankass

		5 to 9-year-olds (N=23,485)	10 to 19-year-olds (N=17,910)
**Ethnicity**	Dogon	21,928 (93.4)	16,709 (93.3)
	Fulani	1,067 (4.5)	830 (4.6)
	Other	490 (2.1)	371 (2.1)
**Wealth quintile**	Wealthiest	5,396 (23.0)	3,683 (20.6)
	Wealthy	4,569 (19.5)	2,928 (16.3)
	Middle	4,509 (19.2)	3,264 (18.2)
	Poor	4,168 (17.7)	3,498 (19.5)
	Poorest	4,783 (20.4)	4,477 (25.0)
	Unknown	60 (0.3)	60 (0.3)
**Decision making contribution of women in household**	Do not contribute	15,568 (66.3)	11,650 (65.0)
	Contribute	7,114 (30.3)	5,664 (31.6)
	Unknown	803 (3.4)	596 (3.3)
**Highest level of reading ability among women in household**	Can read	918 (3.9)	852 (4.8)
	Can partly read	611 (2.6)	489 (2.7)
	Cannot read	21,059 (89.7)	15,908 (88.8)
	Unknown	897 (3.8)	661 (3.7)
**Highest level of schooling among women in household**	Schooling	2,183 (9.3)	1,884 (10.5)
	No schooling	20,497 (87.3)	15,421 (86.1)
	Unknown	805 (3.4)	605 (3.4)
**Polygamy**	Monogamous	11,189 (47.6)	8,370 (46.7)
	Polygamous	11,143 (47.4)	8,587 (47.9)
	Unknown	1,153 (4.9)	953 (5.3)
**Domestic violence**	Not tolerated	5,320 (22.7)	4,028 (22.5)
	Tolerated	17,091 (72.8)	13,057 (72.9)
	Unknown	1,074 (4.6)	825 (4.6)
**Water source**	Improved and treated	4,024 (17.1)	3,211 (17.9)
	Improved but untreated	8,858 (37.7)	6,785 (37.9)
	Unimproved but treated	1,969 (8.4)	1,522 (8.5)
	Unimproved and untreated	8,491 (36.2)	6,276 (35.0)
	Unknown	143 (0.6)	116 (0.6)
**Sanitation**	Improved	11,788 (50.2)	9,241 (51.6)
	Unimproved	11,552 (49.2)	8,544 (47.7)
	Unknown	145 (0.6)	125 (0.7)
**Roofing material**	Natural	766 (3.3)	609 (3.4)
	Rudimentary	2,101 (8.9)	1,610 (9.0)
	Finished	20,506 (87.3)	15,578 (87.0)
	Unknown	112 (0.5)	113 (0.6)
**Wall material**	Natural	7,741 (33.0)	5,868 (32.8)
	Rudimentary	2,348 (10.0)	1,777 (9.9)
	Finished	13,008 (55.4)	9,982 (55.7)
	Unknown	388 (1.7)	283 (1.6)
**Flooring material**	Natural	22,289 (94.9)	17,013 (95.0)
	Rudimentary	350 (1.5)	268 (1.5)
	Finished	815 (3.5)	600 (3.4)
	Unknown	31 (0.1)	29 (0.2)
**Electricity**	Yes	8,515 (36.3)	6,635 (37.0)
	No	14,970 (63.7)	11,275 (63.0)
**Primary cooking fuel**	Wood	19,954 (85.0)	15,144 (84.6)
	Straw	3,109 (13.2)	2,428 (13.6)
	Animal dung	260 (1.1)	191 (1.1)

	Other	162 (0.7)	147 (0.8)
**Food shortage in past 30 days**	Yes	19,961 (85.0)	15,116 (84.4)
	No	3,524 (15.0)	2,794 (15.6)
**Livestock**	Any	21,947 (93.5)	16,988 (94.9)
	Cows/bulls	16,593 (70.7)	13,189 (73.6)
	Horses/donkeys/mules	17,164 (73.1)	13,731 (76.7)
	Goats	11,939 (50.8)	9,550 (53.3)
	Sheep	18,453 (78.6)	14,574 (81.4)
	Chickens	17,285 (73.6)	13,525 (75.5)
**Motorized transport**	Any	12,591 (53.6)	9,719 (54.3)
	Motorbike/scooter	12,546 (53.4)	9,692 (54.1)
	Car/truck	176 (0.7)	136 (0.8)
**Nearest healthcare center, kilometers**	<2	4,289 (18.3)	3,403 (19.0)
	2 - 4.99	6,194 (26.4)	4,553 (25.4)
	5 - 6.99	5,592 (23.8)	3,982 (22.2)
	7 - 9.99	4,599 (19.6)	3,619 (20.2)
	≥10	2,811 (12.0)	2,353 (13.1)

All values are n (%N)

Figure 1 – Probability of death among males and females between ages 5 to 19 years

Table 2 – Age, sex and household characteristics associated with mortality among 5 to 9-year-olds and 10 to 19-year-olds

Characteristic	Categories	Ages 5 to 9-years-old				Categories	Ages 10 to 19-years-old			
		Univariate hazard ratio (95%CI)	p	Multivariate hazard ratio (95%CI)	p		Univariate hazard ratio (95%CI)	p	Multivariate hazard ratio (95%CI)	p
**Age and sex**	Male, 5-7 years	1.00		1.00		Male, 10-14 years	1.00		1.00	
	Male, 8-9 years	0.57 (0.34, 0.95)	0.031	0.57 (0.34, 0.96)	0.033	Male, 15-19 years	1.19 (0.68, 2.08)	0.550	1.16 (0.67, 2.04)	0.594
	Female, 5-7 years	1.00 (0.72, 1.40)	1.000	1.00 (0.72, 1.40)	0.979	Female, 10-14 years	1.07 (0.68, 1.70)	0.766	1.10 (0.70, 1.75)	0.677
	Female, 8-9 years	0.85 (0.51, 1.41)	0.530	0.86 (0.52, 1.42)	0.554	Female, 15-19 years	2.27 (1.28, 4.04)	0.005	2.28 (1.29, 4.02)	0.005
**Ethnicity**	Dogon	1.00				Dogon	1.00			
	Fulani	0.61 (0.21, 1.74)	0.357			Fulani	0.79 (0.29, 2.14)	0.646		
	Other	2.41 (1.25, 4.65)	0.009			Other	0.43 (0.06, 3.04)	0.397		
**Wealth quintile**	Wealthiest	1.00				Wealthiest	1.00		1.00	
	Wealthy	1.68 (1.03, 2.75)	0.037			Wealthy	0.54 (0.23, 1.24)	0.144	0.52 (0.22, 1.19)	0.123
	Middle	1.19 (0.71, 2.00)	0.514			Middle	0.97 (0.48, 1.95)	0.936	0.92 (0.46, 1.85)	0.814
	Poor	1.11 (0.66, 1.85)	0.701			Poor	1.37 (0.72, 2.60)	0.344	1.27 (0.66, 2.43)	0.478
	Poorest	0.98 (0.58, 1.68)	0.951			Poorest	1.79 (1.01, 3.19)	0.048	1.76 (0.99, 3.14)	0.055
	Unknown	-				Unknown	-		-	
**Decision making contribution of women in household**	Contribute	1.00				Contribute	1.00			
	Do not contribute	1.07 (0.76, 1.51)	0.678			Do not contribute	0.98 (0.63, 1.53)	0.937		
	Unknown	-				Unknown	-			
**Highest level of reading ability among women in household**	Can read	1.00				Can read	nc			
	Can partly read	0.77 (0.19, 3.04)	0.706			Can partly read	1.00			
	Cannot read	1.17 (0.52, 2.62)	0.699			Cannot read	1.68 (0.42, 6.81)	0.465		
	Unknown	-				Unknown	-			
**Highest level of schooling among women in household**	Schooling	1.00				Schooling	1.00		1.00	
	No schooling	1.01 (0.60, 1.69)	0.970			No schooling	3.29 (1.21, 8.98)	0.020	3.52 (1.29, 9.62)	0.014
	Unknown	-				Unknown	-		-	
**Polygamy**	Monogamous	1.00				Monogamous	1.00			
	Polygamous	1.15 (0.84, 1.59)	0.375			Polygamous	1.31 (0.86, 1.99)	0.210		
	Unknown	-				Unknown	-			
**Domestic violence**	Not tolerated	1.00				Not tolerated	1.00			
	Tolerated	1.42 (0.94, 2.14)	0.094			Tolerated	0.95 (0.57, 1.59)	0.853		
	Unknown	-				Unknown	-			
**Water source**	Improved and treated	1.00		1.00		Improved and treated	1.00			
	Improved but untreated	1.62 (0.98, 2.67)	0.060	1.61 (0.98, 2.66)	0.061	Improved but untreated	1.41 (0.73, 2.74)	0.307		
	Unimproved but treated	1.13 (0.50, 2.55)	0.766	1.13 (0.50, 2.54)	0.772	Unimproved but treated	2.47 (1.15, 5.32)	0.021		

	Unimproved and untreated	1.83 (1.11, 3.02)	0.017	1.83 (1.11, 3.02)	0.018	Unimproved and untreated	1.70 (0.86, 3.35)	0.126		
	Unknown	-		-		Unknown	-			
**Sanitation**	Improved	1.00				Improved	1.00			
	Unimproved	1.11 (0.81, 1.53)	0.501			Unimproved	0.92 (0.61, 1.39)	0.704		
	Unknown	-				Unknown	-			
**Roofing material**	Finished	1.00				Finished	1.00			
	Rudimentary	0.86 (0.47, 1.59)	0.633			Rudimentary	1.27 (0.64, 2.50)	0.496		
	Natural	1.02 (0.45, 2.29)	0.966			Natural	1.13 (0.42, 3.02)	0.811		
	Unknown	-				Unknown	-			
**Wall material**	Finished	1.00				Finished	1.00			
	Rudimentary	1.62 (0.97, 2.72)	0.064			Rudimentary	1.56 (0.78, 3.15)	0.212		
	Natural	1.34 (0.96, 1.87)	0.088			Natural	1.10 (0.71, 1.72)	0.659		
	Unknown	-				Unknown	-			
**Electricity**	No	1.00				No	1.00			
	Yes	1.02 (0.74, 1.40)	0.917			Yes	0.91 (0.59, 1.39)	0.653		
**Primary cooking fuel**	Wood	1.00				Wood	1.00			
	Straw	1.26 (0.82, 1.94)	0.295			Straw	1.19 (0.71, 2.00)	0.518		
	Animal dung	0.50 (0.07, 3.60)	0.495			Animal dung	1.80 (0.46, 7.10)	0.402		
	Other	0.80 (0.12, 5.41)	0.815			Other	nc			
**Food shortage in past 30 days**	No	1.00				No	1.00			
	Yes	0.79 (0.50, 1.24)	0.300			Yes	1.00 (0.58, 1.73)	0.994		
**Livestock**	No	1.00				No	1.00			
	Yes	0.88 (0.48, 1.60)	0.672			Yes	1.81 (0.58, 5.68)	0.308		
**Motorized transport**	No	1.00				No	1.00			
	Yes	1.12 (0.82, 1.53)	0.485			Yes	0.86 (0.57, 1.28)	0.450		
**Nearest healthcare center, kilometers**	<2	1.00				<2	1.00			
	2 - 4.99	1.40 (0.83, 2.39)	0.211			2 - 4.99	1.07 (0.56, 2.04)	0.832		
	5 - 6.99	1.26 (0.74, 2.15)	0.400			5 - 6.99	0.97 (0.52, 1.83)	0.931		
	7 - 9.99	1.29 (0.75, 2.22)	0.354			7 - 9.99	0.86 (0.44, 1.69)	0.658		
	≥10	1.30 (0.70, 2.41)	0.399			≥10	1.45 (0.75, 2.80)	0.268		

Children and adolescents with missing covariate data were included in analyses but hazard ratios for missing categories are not reported. CI, confidence interval; nc, non-calculable

Table 3 – Marriage, contraception and pregnancy among females aged 15-19 years

		Female 15 to 19-year-olds (N=543)
**Marriage**	Unmarried	390 (71.8)
	Married	147 (27.1)
	Unknown	6 (1.1)
**Age at first marriage, years**	Median (IQR)	18.0 (16.0, 18.0)
	Mean (SD)	17.3 (1.4)
**Contraceptive use***	None	523 (96.3)
	Hormonal contraception	7 (1.3)
	Condom	1 (0.2)
	Other	2 (0.4)
	Unknown	10 (1.8)
**Previously pregnant**	Yes	7 (1.3)
**Currently pregnant**	Yes	6 (1.1)

All values are n (%N) unless otherwise specified. *9 of 10 women using contraception were unmarried. IQR, interquartile range; SD, standard deviation

Figure 1

Click here to access/download;Figure;Figure 1.tif

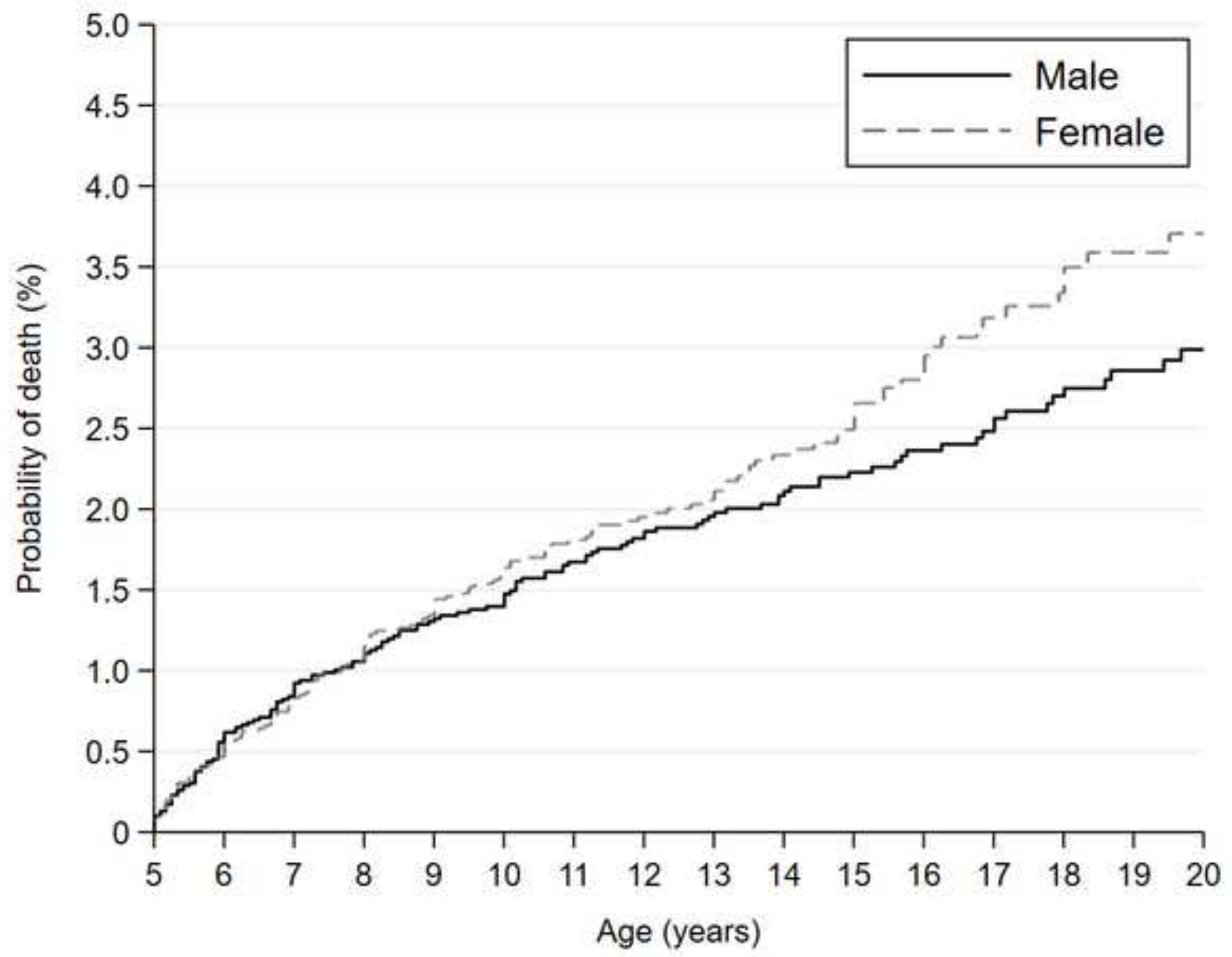

Male n=	6916	6549	5989	5705	5352	5124	4807	4556	4129	3640	3235	2811	2426	2051	1741	1444
Female n=	6458	6138	5664	5395	5024	4932	4414	4019	3562	2929	2367	1933	1586	1206	1016	770

Click here to access/download
Supporting Information
Supplement 20230620.docx

PLOS Global Public Health

30th June 2023

Re: Older child and adolescent mortality in rural Mali

Please see below responses to questions received from the editorial office.

1. Please ensure that the Title in your manuscript and the Title in your online submission form are the same.

All titles we can find appear as "Older child and adolescent mortality in rural Mali". This is the correct title. Apologies if we have misrepresented this somewhere.

2. Please ensure that the Author List in your manuscript file matches the Author List in the online submission form, including initials and order. Currently, authors Jenny Liu and David Charles Boettiger in online submission form but Jenny X. Liu and David C. Boettiger from manuscript.

Author lists in the submission form and manuscript now consistent i.e., David C. Boettiger and Jenny X. Liu

3. If you are submitting a clinical trial, it must be registered in a recognized international registry, e.g. ClinicalTrials.gov or the International Standard Randomized Controlled Trial Number (ISRCTN) Registry. Please include the following with your submission:

1) CONSORT checklist or relevant reporting guideline (uploaded as supporting information)
2) CONSORT flow diagram (uploaded as Fig 1)
3) Trial protocol (uploaded as supporting information).

This is an analysis of baseline data from a clinical trial (i.e., it is a cross-sectional study). Therefore, the STROBE checklist is more appropriate. We have now included a STROBE checklist in our submission.

4. Please complete all items on the Clinical Studies Checklist that are relevant for your submission

Checklist added. Note that, as per item 4 in the checklist, we have added the following: "None of the authors had access to information that could identify individuals during or after data collection." (P6, Line 119)

5. Your study includes child participants, please update your Ethics Statement with confirmation that written informed consent was obtained from the parent/guardian of each participant under 18 years of age.

Despite being an analysis of child and adolescent mortality, our study is based on survey data obtained by interviewing mothers of these children. A statement to this effect is now included in the manuscript: "All women in the household aged 15-49 years (i.e., women of reproductive age) with no plans to leave

The Kirby Institute
Wallace Wurth Building
UNSW Australia
Sydney NSW 2052
T+61 2 9385 0900 F+61 2 9385 0920
E recpt@kirby.unsw.edu.au www.kirby.unsw.edu.au
ABN 57 195 873 179

the study area during the trial period who provided written informed consent were eligible to partake in the baseline survey. No children were surveyed.” (P6, Line 127)

Sincerely

Dr David Boettiger, PhD

The Kirby Institute
Wallace Wurth Building
UNSW Australia
Sydney NSW 2052
T+61 2 9385 0900 F+61 2 9385 0920
E recpt@kirby.unsw.edu.au www.kirby.unsw.edu.au
ABN 57 195 873 179
