# Supplementary material for: Mortality among 5 to 19-year-olds in rural Mali
Source: PLOS Glob Public Health. 2025 Jan 21;5(1):e0004172. doi: 10.1371/journal.pgph.0004172 (PMC11750098; doi:10.1371/journal.pgph.0004172)
Supplement: Attachment — Submitted filename: Response to reviewers 20240724.docx [file pgph.0004172.s007.docx]

***PLOS Global Public Health***

24^th^ July 2024

**Re: Mortality among 5 to 19-year olds in rural Mali (formally: Older child and adolescent mortality in rural Mali)**

Dear Editor

Thank you for the opportunity to resubmit this manuscript to *PLOS Global Public Health*. Please see our responses to editor and reviewer comments below.

Reviewer and Editor comments:

1. (Reviewer) Using of term "Older child": children are defined as individual under 18 years by UNICEF. However, the author uses "older child" to represent those aged 5-9 years, while it may lead to some confusions. I would suggest to use "child" instead of "older child". On the other hand, obviously, the authors have defined the term clearly. So this may be not a problem.

(Editor) At this time, please can you just address the reviewer's concern below regarding the use of the term "older children", which does not seem appropriate for the 5-9 age group. Your title, for example, could simply be more specific about the age range considered in this study.

Response: Thank you. We now refer to “older children” as 5 to 9-year-olds throughout the revised manuscript. Please see the track changes document for further detail.

2. (Reviewer) Table 3 seems not related to other parts of this study. May consider moving to SM.

(Editor) The reviewer's second point is optional.

Response: We believe Table 3 provides important insight into the high mortality reported for adolescent females in Figure 1 and Table 2. We have therefore opted to retain Table 3 in the main document.

Sincerely

Dr David Boettiger, PhD
