# Supplementary material for: Mortality among 5 to 19-year-olds in rural Mali
Source: PLOS Glob Public Health. 2025 Jan 21;5(1):e0004172. doi: 10.1371/journal.pgph.0004172 (PMC11750098; doi:10.1371/journal.pgph.0004172)
Supplement: Attachment — Submitted filename: Response to editorial office 20230630.docx [file pgph.0004172.s005.docx]

***PLOS Global Public Health***

30^th^ June 2023

**Re: Older child and adolescent mortality in rural Mali**

Please see below responses to questions received from the editorial office.

1. Please ensure that the Title in your manuscript and the Title in your online submission form are the same.

All titles we can find appear as “Older child and adolescent mortality in rural Mali”. This is the correct title. Apologies if we have misrepresented this somewhere.

2. Please ensure that the Author List in your manuscript file matches the Author List in the online submission form, including initials and order. Currently, authors Jenny Liu and David Charles Boettiger in online submission form but Jenny X. Liu and David C. Boettiger from manuscript.

Author lists in the submission form and manuscript now consistent i.e., David C. Boettiger and Jenny X. Liu

3. If you are submitting a clinical trial, it must be registered in a recognized international registry, e.g. ClinicalTrials.gov or the International Standard Randomized Controlled Trial Number (ISRCTN) Registry. Please include the following with your submission:

1) CONSORT checklist or relevant reporting guideline (uploaded as supporting information)

2) CONSORT flow diagram (uploaded as Fig 1)

3) Trial protocol (uploaded as supporting information).

This is an analysis of baseline data from a clinical trial (i.e., it is a cross-sectional study). Therefore, the STROBE checklist is more appropriate. We have now included a STROBE checklist in our submission.

4. Please complete all items on the Clinical Studies Checklist that are relevant for your submission

Checklist added. Note that, as per item 4 in the checklist, we have added the following: “None of the authors had access to information that could identify individuals during or after data collection.” (P6, Line 119)

5. Your study includes child participants, please update your Ethics Statement with confirmation that written informed consent was obtained from the parent/guardian of each participant under 18 years of age.

Despite being an analysis of child and adolescent mortality, our study is based on survey data obtained by interviewing mothers of these children. A statement to this effect is now included in the manuscript: “All women in the household aged 15-49 years (i.e., women of reproductive age) with no plans to leave the study area during the trial period who provided written informed consent were eligible to partake in the baseline survey. No children were surveyed.” (P6, Line 127)

Sincerely

Dr David Boettiger, PhD
