# Supplementary material for: Mortality among 5 to 19-year-olds in rural Mali
Source: PLOS Glob Public Health. 2025 Jan 21;5(1):e0004172. doi: 10.1371/journal.pgph.0004172 (PMC11750098; doi:10.1371/journal.pgph.0004172)
Supplement: Attachment — Submitted filename: Response to reviewers 20240211.docx [file pgph.0004172.s006.docx]

***PLOS Global Public Health***

12^th^ February 2024

**Re: Older child and adolescent mortality in rural Mali**

Dear Editor

Thank you for the opportunity to resubmit this manuscript to *PLOS Global Public Health*. Please see our responses to editor and reviewer comments below.

Journal Requirements:

1. We have noticed that you have a list of Supporting Information legends in your manuscript. However, there are no corresponding files uploaded to the submission. Please upload them as separate files with the item type 'Supporting Information'.

Response: We have now uploaded all Tables and Figures as separate files, as well as our Supplementary Material.

Reviewer #1:

1. The introduction of the article effectively sets the context and highlights the research gap concerning older children and adolescents in LMICs. It emphasizes the significance of studying this population given their role in shaping future health and well-being. The introduction provides a comprehensive overview of the existing literature and the specific issues related to this age group in the study area, which is crucial for understanding the context of the research.

Response: Thank you for this positive feedback.

2. The methods section is detailed and well-structured, providing a clear explanation of the data sources, inclusion criteria, variable definitions, and statistical analysis. The authors have justified their approach to conducting the research, including the use of household survey data and the rationale for analyzing older children and adolescents separately. However, there are a few points to consider for clarification and improvement:
   - The authors mention "lifetime birth histories" without explaining what this entails. It would be helpful to provide a brief description or reference.

Response: We have now added a definition for further clarity. The definition reads: “Lifetime birth history refers to the number of births a woman has had in her life, the dates they occurred, and their outcome.”

- While the article mentions ethical approval, it would be beneficial to provide more details about the ethical considerations, especially when working with sensitive data.

Response: We appreciate this important point. The Ethics approval and consent to participate section now includes the wording: “The main consideration for this analysis was the potential for data access permitting identification of study participants. The University of California, San Francisco exempted secondary analysis of the deidentified trial data from ethical approval based on the thorough deidentification of participants.”

- The article discusses the use of global positioning technology for geographic coordinates but does not provide information on how this data was collected and utilized in the analysis.

Response: Thank you – we have now clarified the collection and use of GPS data. The revised sentence reads: “Household distance to the nearest healthcare center was determined using orthodromic (great circle) distance estimates between family compound and healthcare center GPS coordinates.”

3. The presentation of results is clear and well-organized. The use of Kaplan-Meier curves to visualize mortality trends is effective. The tables and figures provide a concise summary of the findings. However, there are a few areas that could be improved:

- The discussion of the reversal in mortality trends between males and females during adolescence is intriguing, but the article does not delve into potential explanations or implications. It would be beneficial to include some speculation or hypotheses regarding this observation.

Response: Thank you – we refer the reviewer to the revised third paragraph of the Discussion where the reversal in mortality trend among males and females during adolescence is discussed in detail:

“This trend reversal and disparity underscores the importance of the confluence of risk factors, such as early marriage, childbearing, access to reproductive health, and gender-based violence, among many other inter-related dimensions of gender inequities (e.g., literacy, education, economic and financial dependence) that uniquely affect access to healthcare and directly increase mortality risk among adolescent girls and young women in LMICs ^26^. Accordingly, we also find that low maternal education as a unique driving risk factor among females. This may be indicative of gender inequalities that disadvantage women. Our data do not permit a more detailed analysis of individual-level risk factors associated with female adolescent mortality, a limitation for understanding the significance of this result. Further, in comparison to Mali nationally, a lower percentage of females 15-19 in Bankass were married (27.1% vs. 39.7%), a surprising finding given the extreme poverty and poor health indicators for the region which are often strongly associated with early marriage. However, our sample of females age 15-19 in Bankass is small, as a larger number of older adolescent females than males were reported by their household to migrate out of the study area, which may be for reasons of marriage or employment, and also limits measurement accuracy. More research with a larger, in-depth survey of individuals is needed to fully assess and disentangle these risk factors for both males and females in our study population.”

- While the results highlight the higher risk of mortality among adolescents in poorer households and those with lower maternal education, the article does not explore the potential mechanisms or pathways through which these factors influence mortality. Adding some discussion on these aspects could enhance the interpretation of the findings.

Response: Thank you for this suggestion. We have added further discussion around potential pathways in the second paragraph of the Discussion. We believe this – combined with the revised third paragraph included in the above response - adds greater insight into our findings. The revised paragraph reads:

“Notably, the risk of mortality is especially acute among female adolescents compared to their male counterparts. Known indicators of poverty such as poor infrastructure and low wealth- which increase mortality by limiting access to appropriate healthcare - contributed overall. Low maternal education within the household was a uniquely contributing factor for females. This difference in mortality by sex reverses the trend observed in our earlier analysis of under-fives in which mortality among males was greater than females ^17.^ Previous work has shown that biological mechanisms lead to greater mortality in males among under-fives^23,24^. Of available global average trends, including among high-income countries and LMICs, the risk of death among males increases overall and in relation to females during adolescence; only within African and Southeast Asian countries do females experience greater mortality into young adulthood ^25^, similar to what we observe in rural Mali.”

4. The discussion section effectively addresses the implications of the research and highlights the need for further studies in this area. However, there is room for improvement in the following areas:
   - The discussion could benefit from a more in-depth exploration of the potential causes of the observed mortality trends, especially the higher risk among female adolescents. Speculation or hypotheses regarding the role of early marriage, gender-based violence, or other factors could add depth to the analysis.

Response: This is a very similar point to that made in part 1 of this reviewer’s third point. Please see our response to that comment above.

- The limitations of the study are mentioned but could be expanded upon. For example, the potential sources of bias, such as recall bias in reporting deaths, should be discussed in more detail.

Response: Thank you. We have now created a separate Limitations section between the Discussion and Conclusion. This section points out the potential impact of recall bias on our findings. The Limitations section now reads: “Our findings should be interpreted considering several additional limitations. Mortality was based on household members’ recall of deaths in the preceding five years, which may be subject to recall errors, particularly for deaths occurring early in the five-year period. This may have led to a slight under- or overestimate of mortality rates. Elucidation of individual and household risk factors were further limited by the scope of survey questions, which do not represent a comprehensive assessment, particularly for individual-level factors (e.g., physical violence) that other studies have found to importantly contribute to older child and adolescent mortality 7,8. Finally, we are unable to further ascribe the causes and contributors to mortality, which may require more in-depth verbal autopsy approaches in the absence of vital registration data”

- The authors should consider including a section on policy implications or recommendations based on their findings - "How can the results inform health programming and policies in LMICs, particularly in rural Mali?".

Response: This point is also made by Reviewer 2. We thank both reviewers for this helpful suggestion. Our revised conclusion now reads: “Limitations notwithstanding, our data is unique among population health surveys in its comprehensive census of a large population in a resource-poor area of the world with one of the highest burdens of child mortality. Our findings emphasize the importance of ensuring policy and program advances in rural Mali and similar parts of sub-Saharan Africa address the needs of older children and adolescents. Interventions focused on improving household infrastructure and maternal education may be especially impactful. Our results also point to the critical need for further research and health programming to improve survival among older children and adolescents—in particular, a better accounting and measurement of their health outcomes and associated risk factors. The lack of attention to this gap does little to improve global health equity and must be urgently addressed, beginning with inclusion of these groups in population health research.”

Reviewer #2:

1. This manuscript is an important contribution to knowledge and understanding of mortality in older children and adolescents. In general, the manuscript is well-written and comprehensible.

However, I suggest that you copy edit the manuscript before resubmitting it. Many sentences and paragraphs are overly long, making it difficult to understand them without re-reading. In addition, there are several instances of unclear phrasing, as I have highlighted in the attachment.

Response: We thank the reviewer for their positive feedback on our work. We have been over the manuscript again and agree there were a number of sentences that needed to be made clearer. We have made various corrections, including those suggested by the reviewer in their attachment. We are very appreciative of the reviewer’s detailed review.

I suggest that you create a separate section for the limitations of the study.

Response: We have now created a sub-titled Limitations section between the Discussion and the Conclusion.

In addition, your conclusion focuses only on the dearth of research on the topic of older children and adolescents. I suggest strengthening your conclusion to include how the findings of this study can be used to inform policy and programs in rural Mali and beyond.

Response: Please see response to Reviewer 1’s fourth question (part 3).

Sincerely

Dr David Boettiger, PhD
