# Supplementary material for: Mortality among 5 to 19-year-olds in rural Mali
Source: PLOS Glob Public Health. 2025 Jan 21;5(1):e0004172. doi: 10.1371/journal.pgph.0004172 (PMC11750098; doi:10.1371/journal.pgph.0004172)
Supplement: Attachment — Submitted filename: Response to reviewers 20241106.docx [file pgph.0004172.s008.docx]

***PLOS Global Public Health***

6^th^ November 2024

**Re: Mortality among 5 to 19-year olds in rural Mali (formally: Older child and adolescent mortality in rural Mali)**

Dear Editor

Thank you for the opportunity to resubmit this manuscript to *PLOS Global Public Health*. Please see our responses to the reviewer comments below.

Reviewer comments:

1. I enjoyed reviewing this manuscript. The authors used data from randomized controlled trial to estimate mortality rates and assess risk factors of mortality among 5-9 years old and adolescents in rural Mali. The paper is well-written, clear, and certainly worthy of publication. I also saw that the authors have taken great care to address the comments by previous reviewers. A minor concern that I would like the authors to address: Variables that are conceptually similar – In standard surveys, ‘wealth quintile’ is typically measured using a principal component analysis of several indicators of household wealth, such as drinking water source, roofing material, flooring material, toilet facilities, electricity, primary cooking fuel, and ownership of livestock. In the current manuscript, a number of independent variables are somewhat similar conceptually i.e., wealth quintile as compared to water source, roofing material, wall material, flooring material, electricity, primary cooking fuel, and livestock. My question is, should water source, roofing material etc. be included in the analytic models since these variables were used to create wealth quintile? In my opinion, it is redundant including variables that represent the same phenomenon, even if they have different names. Including conceptually similar variables in a study is generally not recommended, but if the authors must include them, I think a justification is warranted. To address this concern, the authors can do one or both of the following: (a) clarify whether some of the variables used in creating wealth quintile are also included as independent variables, and maybe provide a justification; (b) examine the association/ correlations among these somewhat similar variables using appropriate statistical tests. If strongly associated, maybe include only the most relevant variable(s). Overall, this paper is a valuable contribution and I hope to see it published following revisions.

Response: Thank you for the kind words. Measuring wealth index with a principal components analysis in a resource-limited setting (such as Bankass) is akin to measuring household income in a higher income setting. Although the wealth index uses a range of household characteristics to define wealth (some of which are included as independent variables in our regression analyses), it is not a sufficient indicator of each of these characteristics independently. Hence, it is appropriate to include wealth index along with variables used to define wealth index in the same model. Reassuringly, we did not find collinearity among any of the variables included in our final models. Please see relevant additions to the revised manuscript below:

From the Methods section – *“Multicollinearity was evaluated by calculating variance inflation factors for each covariate included in our final models.”*

From the Results section for Factors associated with death in 5 to 9-year-olds, and Factors associated with death in adolescents (ages 10-19 years) – *“All covariates included in our overall and sex-specific final models had a variance inflation factor <2 indicating multicollinearity was not an issue.”*

Sincerely

Dr David Boettiger, PhD
